# Supplementary material for: Genome-Wide Identification and Characterization of the Abiotic-Stress-Responsive GRF Gene Family in Diploid Woodland Strawberry (Fragaria vesca)
Source: Plants (Basel). 2021 Sep 15;10(9):1916. doi: 10.3390/plants10091916 (PMC8468544; doi:10.3390/plants10091916)
Supplement: Supplementary file 1 [file plants-10-01916-s001.zip › plants-1374032-supplementary/Supplementary materials.pdf]

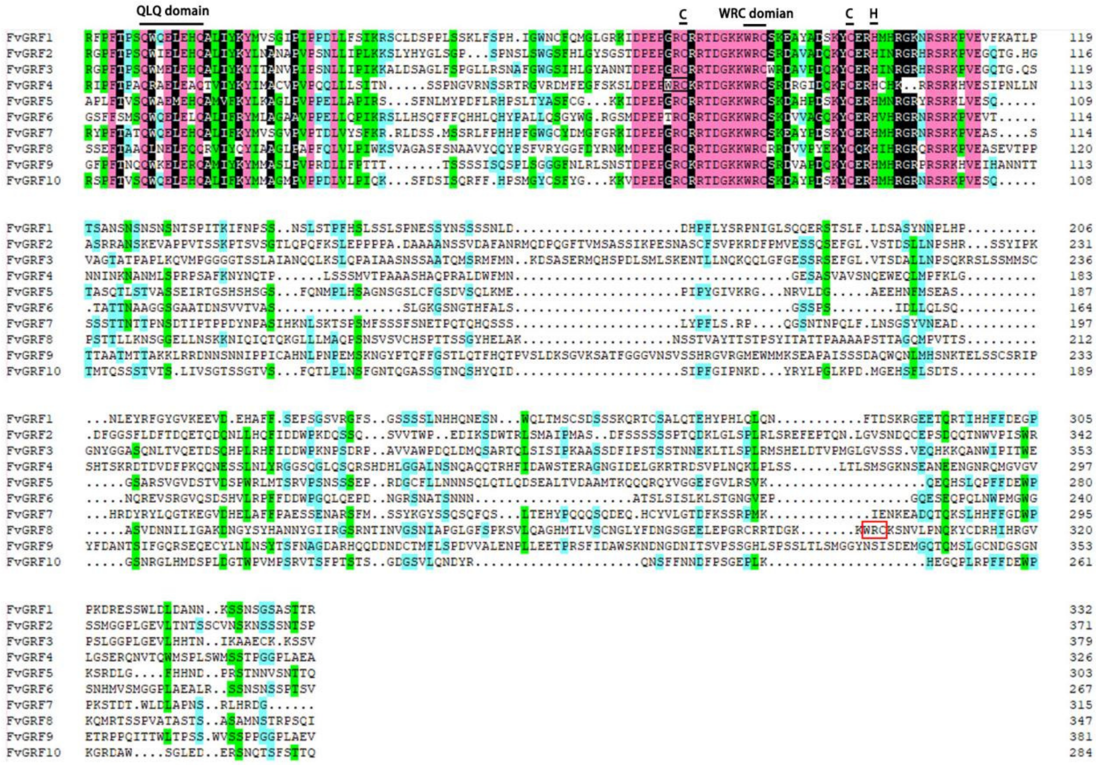

Figure S1. Sequence alignment of FvGRF (Fragaria vesca GRF) proteins and the QLQ and WRC domains are indicated upside. Identical amino acids are indicated by the color background.

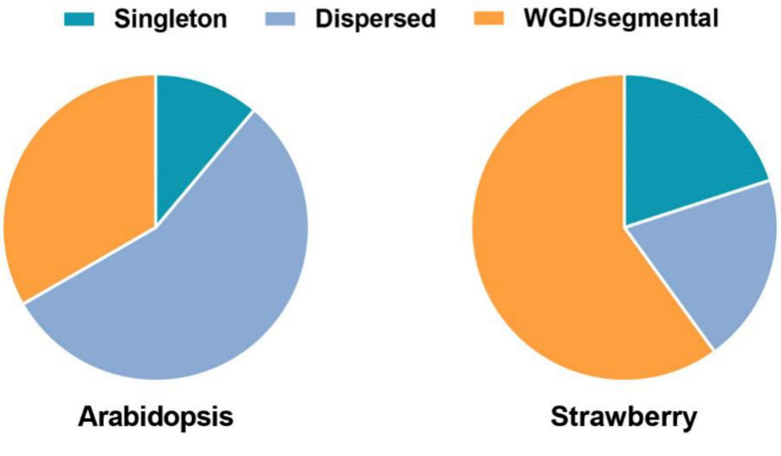

Figure S2. Relative frequency of different modes of gene duplication in *Arabidopsis thaliana* and woodland strawberry. Different colors represent different types of gene duplication.
